# Incidence of unintended pregnancy and associated factors among adolescent girls and young women at risk of HIV infection in Kampala, Uganda

**DOI:** 10.3389/frph.2023.1089104

**Published:** 2023-02-23

**Authors:** Mary Namukisa, Onesmus Kamacooko, Jane Frances Lunkuse, Eugene Ruzagira, Matt A. Price, Yunia Mayanja

**Affiliations:** ^1^ Department of Global Health Collaborative, Mbarara University of Science and Technology, Mbarara, Uganda; ^2^ Department of Data and Statistics, Medical Research Council/ Uganda Virus Research Institute and London School of Hygiene and Tropical Medicine (MRC/UVRI & LSHTM) Uganda Research Unit, Entebbe, Uganda; ^3^ Department of HIV Epidemiology and Intervention, Medical Research Council/Uganda Virus Research Institute and London School of Hygiene and Tropical Medicine (MRC/UVRI & LSHTM) Uganda Research Unit, Entebbe, Uganda; ^4^ Department of Infectious Disease Epidemiology, London School of Hygiene and Tropical Medicine, London, United Kingdom; ^5^ Department of Epidemiology, IAVI, New York, NY, United States; ^6^ Department of Epidemiology and Biostatistics, University of California, San Francisco, San Francisco, CA, United States

**Keywords:** pregnancy, adolescents, young women, sub-Saharan Africa, contraception

## Abstract

**Background:**

In sub-Saharan Africa, one in every five young women becomes pregnant, and 50% of these are unintended. Pregnancies in adolescent girls and young women (AGYW) are associated with poorer maternal and neonatal outcomes and a high abortion rate, yet data are still limited on incident pregnancies among AGYW in vulnerable situations. We studied the incidence and factors associated with unintended pregnancy among AGYW who were frequently engaged in transactional sex in Kampala, Uganda.

**Methods:**

We analyzed data from a study that investigated the uptake of oral pre-exposure prophylaxis among AGYW from January 2019 to December 2020. Volunteers attended 3-monthly study visits for 12 months each. Contraceptive services were provided to interested volunteers free of charge. Interviewers collected data on sociodemographics, sexual behavior, reproductive health outcomes, and substance use. Pregnancy was determined by testing for beta-human chorionic gonadotropin hormone in urine. The pregnancy incidence rate was estimated using the Kaplan–Meier technique, and logistic regression was used to determine the correlates of pregnancy.

**Results:**

We included 285 volunteers with a mean age of 19.9 [standard deviation (SD), ± 2.24] years; 54.7% had attained secondary school education or higher, 57.2% were single (never married), 92.6% reported engaging in transactional sex, 21.0% reported sex work as their main job, 51.9% consumed alcohol in the month prior to the interview, of whom 12.8% consumed alcohol daily, and 25.3% had *Chlamydia trachomatis*/*Neisseria gonorrhoeae*. The mean age at first sexual intercourse was 15.7 (SD, ±2.1) years. We recorded 44 pregnancies over 187.2 person-years of follow-up, an incidence of 23.5 per 100 person-years [95% confidence interval (CI), 17.5–31.6]. Incident pregnancies were more likely among volunteers who had ≥10 sexual partners in the past 3 months [adjusted risk ratio (aRR) 1.97; 95% CI, 1.05–3.70] and those who reported not using contraception (aRR 5.89; 95% CI, 2.74–12.66). Incident pregnancies were less likely among those who reported alcohol consumption in the past month (aRR 0.52; 95% CI, 0.30–0.90).

**Conclusion:**

The incidence of unintended pregnancy was high despite the availability of free contraceptive services. We recommend sociobehavioral studies to explore this further. Sexual and reproductive health campaigns should strengthen demand creation and motivation to use contraception among young women with multiple sexual partners.

## 1. Introduction

In 2016, the World Health Organization (WHO) released strategies toward “Ending Preventable Maternal Mortality,” where meeting the need for family planning and reducing adolescent birth rate were set among core indicators (1, 2). Additionally, target 3.1 of sustainable development goal (SDG) 3 to “*ensure healthy lives and promote well-being for all at all ages*” aims at reducing the maternal mortality ratio to less than 70 for every 100,000 live births by 2030 (3). In 2020, the global maternal mortality ratio was 152 deaths per 100,000 live births, with sub-Saharan Africa (SSA) having the highest ratio at 302 deaths per 100,000 live births, followed by South Asia at 147 deaths per 100,000 live births (4). According to WHO, young people below the age of 20 years account for 11% of childbirths globally, 95% of which are from low- and middle-income countries (LMICs). Additionally, in LMICs, at least 10 million unintended pregnancies occur each year among young women below the age of 20 years (5). Worldwide, there has been an increase in both the availability and awareness of contraception among women of reproductive age (6, 7). However, in LMICs like Uganda, the rate of unintended pregnancies remains a big challenge and was reported to be 64% among women aged 15–49 years between 2015 and 2019, with 39% of these pregnancies ending in abortion (8).

Unsafe abortions and complications during pregnancy and childbirth are the leading cause of mortality among girls aged 15–19 years, and countries with abortion restrictions have had an increase in the proportion of unintended pregnancies ending in abortion over the years (9). In 2021, SSA, Latin America, and the Caribbean registered the highest rates of adolescent birth rates, while South Asia showed a great decline (5, 10). A systematic review and meta-analysis of studies among adolescents in SSA showed that one in every five adolescents becomes pregnant, with East Africa having the highest prevalence (21.5%) of adolescent pregnancies (11). In 2016, it was estimated that about 50% of adolescent pregnancies in LMICs were unintended, which contributed to a high abortion rate (12, 13). Furthermore, unintended pregnancies have been associated with poorer neonatal and maternal outcomes among adolescents and younger women, including but not limited to low birth weight, abortions, stillbirth, and neonatal death (14). Access to modern contraception has been identified as a strategy to achieve SDG 3 (15), given its positive effect on economic growth, environmental protection, and poverty reduction (16) and its impact on reducing both neonatal and maternal mortality (17).

In East Africa, the unmet need for contraception among adolescents aged 15–19 years was higher than that among all women of reproductive age (13). A report from the Naguru teenage center in Kampala, Uganda, showed that 40% of adolescents who visited the teenage center were pregnant and adolescents with no education were more likely to become pregnant compared to those with formal education (18). Amongin et al., in another study done in Uganda, showed a 16% decrease in the proportion of pregnant adolescents between 2000 and 2016 and an increase in the demand to delay pregnancy between 1988 and 2016 (19). Studies on young women in Uganda and Tanzania reported that unintended pregnancies are associated with no or low education, younger reported age at sexual debut, being married previously, and an increased number of sexual partners (19, 20). In other SSA settings, adolescent pregnancy has been associated with no maternal or paternal education and a lack of parent-to-adolescent communication on sexual and reproductive health issues (11).

Despite efforts to make modern contraceptives accessible for all women of reproductive age, the United Nations Population Fund reports that only 49% of sexually active young girls in Uganda used a modern contraceptive method during their last sexual encounter (21). Few studies have followed up with young women in SSA who are behaviorally vulnerable to pregnancy and HIV infection to report on the incidence of unintended pregnancies, and yet pregnancies among young people are more likely to occur in marginalized communities, commonly driven by poverty and a lack of education and employment opportunities (22). In this study, we present the incidence and factors associated with unintended pregnancies among AGYW in Kampala, Uganda, who frequently reported transactional sex.

## 2. Methodology

### 2.1. Study design and setting

We analyzed data from a 2-year cohort study done among AGYW. The study was conducted at the Good Health for Women Project (GHWP) clinic, which was established in 2008 in a periurban community in southern Kampala (23)**.** The clinic provided free general healthcare that included HIV prevention and treatment services, reproductive healthcare, and free family planning methods, including injectables, implants, and combined oral contraceptives to eligible women.

### 2.2. Study population, sampling, and eligibility

The study population included AGYW aged 14–24 years who were enrolled in the “*Interventions for HIV Prevention among Adolescent girls and young women (IPAD)*,” whose overall aim was to assess knowledge and preferences for biomedical HIV prevention interventions and uptake of oral pre-exposure prophylaxis among AGYW in Kampala, Uganda. The IPAD study therefore recruited HIV-negative AGYW who reported risk behavior that made them eligible for oral pre-exposure prophylaxis (PrEP). Recruitment and follow-up of study volunteers from urban slums through project field workers and AGYW peers were done from January 2019 to December 2020, and the study has been described by Mayanja et al. (24), including enrollment of 14–17 year olds only if they were identified as emancipated and/or mature minors according to national guidance (25). These minors could consent for themselves without parental/guardian approval. We included volunteers who were not pregnant at enrollment and were willing to use an effective contraception method during the study.

### 2.3. Data collection

Research nurses collected data at 3-monthly study visits using interviewer-administered questionnaires. Data were collected on sociodemographic characteristics, sexual behavior, contraceptive use, pregnancy, sexually transmitted infections (STIs), and substance use. Volunteers who became pregnant were linked to antenatal care services and followed up for a pregnancy outcome.

Laboratory tests included pregnancy tests for beta-human chorionic gonadotrophin (β-hcg) hormone performed on urine samples using QuickVue (Quidel Corporation, San Diego, CA, United States) and HIV tests following the national testing algorithm, which involves a screening test (Alere Medical Co. Ltd., Chuba, Japan), the StatPak rapid confirmatory kit (Chembo Diagnostics System Inc., Medford, NY, United States), and SD Bioline as a tiebreaker (Standard Diagnostics, Inc., South Korea). STIs (*Neisseria gonorrhoeae* and *Chlamydia trachomatis*) were tested on endocervical swabs using the GeneXpert platform (Cepheid AB, Rontgenvagen 5, Soina, Sweden).

### 2.4. Measurement of variables

#### 2.4.1. Primary outcomes

The primary outcome was the first incident pregnancy during study follow-up for an enrolled volunteer who tested negative for pregnancy at the previous study visit.

#### 2.4.2. Independent variables

Sociodemographic variables included age at enrollment, marital status, education level, main job/ employment, weekly income, and the number of biological children.

Behavioral and reproductive health variables included age at first sexual intercourse and the following variables collected for the past 3 months: number of sexual partners, paid sex (yes/no), group sex (yes/no), forced sex (yes/no), contraceptive use (yes/no), having an STI (*Chlamydia trachomatis*/*Neisseria gonorrhoeae*), frequent travel from home, i.e., traveling for ≥3 nights away from home per week (yes/no), and illicit drug use (yes/ no). Screening for harmful alcohol consumption was done using the Alcohol Use Disorders Identification Test (AUDIT). AUDIT scores were categorized as follows: 0–7, low risk; 8–15, moderate risk/hazardous; ≥16, high risk.

### 2.5. Statistical analysis

Data were double-entered in OpenClinica, cleaned, and exported to STATA 17.0 (StataCorp, College Station, TX, United States) for analysis. Descriptive analysis was used to understand baseline group characteristics. We determined the proportion that became pregnant as the number that tested positive for pregnancy during study follow-up divided by the total number of participants who tested negative for pregnancy at enrollment (i.e., 100% of our study population, by design). To estimate the incidence of pregnancy, we used the Kaplan–Meier technique to estimate the time to test positive for pregnancy after enrollment into the study and presented the estimated incidence rate per 100 person-years. To estimate baseline factors associated with incident pregnancy, we used Poisson regression analysis with robust error variance both for unadjusted and adjusted levels; variables for which the association attained *p* < 0.15 at the unadjusted level were selected for the multivariable model except for age, which was considered an *a priori* confounder. We tested for collinearity between the baseline audit score and the question “have you had any alcoholic drink in the past month?” The results showed no collinearity (variance inflation factors <5). However, due to the very small number of participants with the outcome for the high-risk alcohol use category, we did not consider the baseline audit score for the multivariable model. Variables with a *p*-value <0.05 at multivariable analysis were considered significantly associated with incident pregnancy. Results are presented as adjusted risk ratios (aRR) with *p*-values and 95% confidence intervals (CIs).

### 2.6. Ethical considerations

The study was approved by Uganda National Council for Science and Technology (HS 2435) and Uganda Virus Research Institute Research Ethics Committee (GC/127/18/06/658). All volunteers gave written informed consent before study participation**.** The study enrolled emancipated and/or mature minors (14–17 years) who consented for themselves according to national guidelines for the enrollment of emancipated and/or mature minors.

## 3. Results

A total of 285 volunteers with a mean age 19.9 [standard deviation (SD),±2.24] years were included in the analysis. Of these, 60.7% were aged 20–24 years, 54.7% had attained secondary education and above, and 57.2% were single (never married) (Table 1). The mean age at first sexual intercourse was 15.7 (±SD, ±2.1) years. We observed that 92.6% reported engaging in transactional sex in the past 3 months; however, only 21.0% reported sex work as their main job. Volunteers who did not report engaging in sex work as their main occupation had other primary jobs, including working in hospitality, e.g., restaurants, guest houses, beauty salons (3.2%), entertainment facilities, e.g., night clubs and bars (26.3%), and several other main jobs (25.6%); 23.9% reported being unemployed. One hundred forty-eight volunteers (51.9%) reported to have consumed alcohol in the last month, of whom 12.8% took alcohol daily, 42.6% took alcohol weekly, and 44.6% took alcohol 1–3 times a month. Contraceptive use at baseline was higher among volunteers who consumed alcohol in the past month than those who did not (59.5% vs. 40.5%, *p* = 0.004). Additionally, 21.9% were screened as moderate to high-risk alcohol drinkers on the AUDIT tool. The baseline characteristics of volunteers are given in Table 1.

**Table 1:** Baseline sociodemographic and behavioral characteristics of adolescent girls and young women by incident pregnancy status.

Volunteer baseline characteristics	Got an incident pregnancy (*N* = 44) *n* (row %)	Did not get an incident pregnancy (*N* = 241) *n* (row %)	Overall (*N* = 285) *n* (col %)
Age at enrollment (years)			
14–19	21 (18.8)	91 (81.2)	112 (39.3)
20–24	23 (13.3)	150 (86.7)	173 (60.7)
Education level			
Below secondary level	20 (15.5)	109 (84.5)	129 (45.3)
Secondary level and above	24 (15.4)	132 (84.6)	156 (54.7)
Marital status			
Ever been married	19 (15.6)	103 (84.4)	122 (42.8)
Single (never married)	25 (56.8)	138 (57.3)	163 (57.2)
Number of biological children			
None	18 (17.5)	85 (82.5)	103 (36.1)
One child or more	26 (14.3)	156 (85.7)	182 (63.9)
Main job			
Sex work	7 (11.7)	53 (88.3)	60 (21.1)
Hospitality and entertainment	19 (22.6)	65 (77.4)	84 (29.5)
No job/unemployed	10 (14.7)	58 (85.3)	68 (23.9)
Other jobs	8 (11.0)	65 (89.0)	73 (25.6)
Age at first sexual intercourse (years)			
<15	15 (18.1)	68 (81.9)	83 (29.1)
≥15	29 (14.4)	173 (85.6)	202 (70.9)
Consumed alcohol in the last month			
No	28 (20.4)	109 (79.6)	137(48.1)
Yes	16 (10.8)	132 (89.2)	148 (51.9)
Had sex while drunk in the last month			
No	33 (17.4)	157 (82.6)	190 (66.7)
Yes	11(11.6)	84 (88.4)	95 (33.3)
Number of sexual partners in the past 3 months			
1 partner	14 (15.7)	75 (84.3)	89 (31.2)
2–9 partners	19 (13.5)	122 (86.5)	141 (49.5)
10+ partners	11 (20.0)	44 (80.0)	55 (19.3)
Coerced sex in the past 3 months			
Yes	10 (14.9)	57 (85.1)	67 (23.5)
No	34 (15.6)	184 (84.4)	218 (76.5)
Currently using contraception			
Yes	7 (4.6)	146 (95.4)	153 (53.7)
No	37 (28.0)	95 (72.0)	132 (46.3)
Paid sex in the past 3 months			
Yes	42 (15.9)	222 (84.1)	264 (92.6)
No	2 (9.5)	19 (90.5)	21 (7.4)
Group sex in the past 3 months			
Yes	5 (15.6)	27 (84.4)	32 (11.2)
No	39 (15.4)	214 (84.6)	253 (88.8)
Frequent travel from home in the last 3 months^a^			
Yes	17 (14.9)	97 (85.1)	114 (40)
No	27 (15.8)	144 (84.2)	171 (60)
AUDIT score			
Low-risk drinker	40 (19.8)	162 (80.2)	202 (70.9)
Moderate to high-risk drinker	4 (4.8)	79 (95.2)	83 (21.9)
Had an STI (chlamydia or gonorrhea)^b^			
Yes	6 (15.0)	34 (85.0)	40 (14.2)
No	38 (15.7)	204 (84.3)	242 (85.8)

AUDIT, Alcohol Use Disorders Identification Test; STI, sexually transmitted infection.

^a^ Frequent travel = ≥3 nights away from home per week.

^b^ Three volunteers were not tested for STIs.

### 3.1. Pregnancy incidence during the study

**Figure 1:**
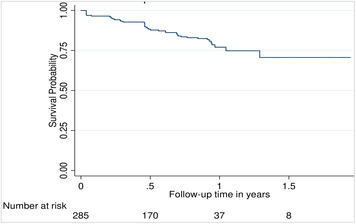
Incidence of pregnancy among adolescent girls and young women during study follow-up (2019–2020).

The median follow-up time per volunteer was 0.90 years (IQR 0.22–0.94 years). We recorded a total of 44 incident pregnancies over 187.2 person-years of follow-up, an estimated incidence rate of 23.5 per 100 person-years (95% CI, 17.5–31.6). Of the 44 participants, 37 were not using contraception at baseline. We observed three volunteers who tested positive for pregnancy a second time (these three pregnancies are not considered in our calculation of “incident pregnancy”). No volunteer tested positive for pregnancy more than twice. The outcomes of the 47 total pregnancies (includes 3 repeat pregnancies) included 20 live births (1 preterm), 3 still births, and 11 abortions (2 spontaneous). Thirteen volunteers had no documented outcome because they could not be reached. Details of incident pregnancy after enrollment into the study are shown in Figure 1.

### 3.2. Baseline factors associated with incident pregnancy during the study

In the adjusted model, participants who reported consuming alcohol in the last 1 month were less likely to become pregnant compared to those who reported not consuming alcohol (aRR 0.52; 95% CI, 0.30–0.90), while participants who had ≥10 sexual partners were more likely to become pregnant compared to those with one sexual partner (aRR 1.97; 95% CI, 1.05–3.70). The results further show that those who were not using contraception were more likely to become pregnant than those who used contraception (aRR 5.89; 95% CI, 2.74–12.66). Details are presented in Table 2.

**Table 2:** Baseline factors associated with becoming pregnant among adolescent girls and young women in Kampala, Uganda.

Baseline characteristics	uRR (95% CI)	*p*-value	aRR (95% CI)	*p*-value
Age at enrollment (years)				
14–19	Ref		Ref	
20–24	0.57 (0.33–0.98)	0.045	0.85 (0.51–1.42)	0.538
Number of biological children				
None	Ref			
One child or more	0.82 (0.47–1.42)	0.474	–	
Number of sexual partners in the past 3 months				
1	Ref		Ref	
2–9	0.86 (0.45–1.62)	0.635	0.92 (0.51–1.67)	0.79
10+	1.27 (1.02–3.84)	0.049	**1.97 (1.05–3.7)**	**0.034**
Main job				
Sex work	Ref			
Hospitality	1.94 (0.87–4.32)	0.106	–	
No job	1.26 (0.51–3.11)	0.615	–	
Other	0.94 (0.36–2.45)	0.898	–	
Consumed alcohol in the last month				
No	Ref		Ref	
Yes	0.53 (0.3–0.93)	0.028	**0.52 (0.3–0.9)**	**0.019**
Currently using contraception				
Yes	Ref		Ref	
No	6.13 (2.82–13.3)	<0.001	**5.89 (2.74–12.66)**	**<0.001**

Bold values represent statistically significant results. uRR, unadjusted risk ratio; aRR, adjusted risk ratio; CI, confidence interval; Ref, reference.

## 4. Discussion

In this study conducted to determine the incidence of unintended pregnancy and associated factors among AGYW at risk of HIV infection, we recorded a high incidence of pregnancy, with nearly a quarter of our participants becoming pregnant annually. Contrary to our findings, lower incidence rates of unintended pregnancy have been reported from surveys done among 15–19 year olds in Zimbabwe (26) and Rakai in Western Uganda (27). In the study done in Zimbabwe, an incidence of 9% was reported from a cross-sectional analysis (August to November 2016) that included data from three nationwide surveys of postabortion care facilities, health professionals as key informants, and postabortion care patients (26). This study included a diverse group of young people who differed by sociodemographic and other characteristics, including those with less frequent sexual exposure compared to most of our study volunteers who reported engaging in transactional sex. This likely explains the lower incidence found by others. Furthermore, a 6% incidence was reported from an analysis of study data collected over 20 years (1994–2013) in the Rakai community cohort, Uganda (27). During the latter half of this period, antiretroviral therapy (ART) was rolled out in the country. The incidence and prevalence of orphanhood therefore reduced over time as mortality from AIDS declined in a region among the worst hit regions by the epidemic in Uganda (28). Santelli et al. further reported that socioeconomic status and school enrollment also improved in Rakai after the ART rollout period. School enrollment was protective for adolescents as it was associated with higher contraceptive use and fewer sexual partners (27). School enrollment has been associated with lower teenage pregnancy rates elsewhere in SSA (29, 30) and is enabled by structural factors such as the supervised school environment, education obtained, and safer peer networks. Regarding studies done on adolescents living with HIV, much lower incidences have been reported (1.8%–2.2%) (31, 32). This is likely due to regular interface with ART providers where sexual reproductive health services are offered as part of comprehensive HIV care and treatment and emphasis is given on the second prong of elimination of mother-to-child transmission of HIV (EMTCT) “*preventing unintended pregnancies among women living with HIV*.” This has a role in increasing contraceptive use and subsequently reducing pregnancy rates. Indeed, cost-effective analyses have shown that contraceptive strategies to prevent unintended pregnancies among HIV-positive women would avert 28.6% more HIV-positive births than nevirapine for EMTCT (33).

Although half of the AGYW reported using an effective contraceptive method at baseline, we observed that four out of every five volunteers who became pregnant were not using contraception at baseline. The strong relationship between the nonuse of contraception and incident pregnancy that we report has also been seen among AGYW elsewhere and in the general population (34, 35). We enrolled young women; young age has been associated with increased risk of incident pregnancy (36), and our findings could be explained by participants’ frequent involvement in sexual behavior (e.g., multiple sexual partnerships, paid sex), which increased their likelihood of becoming pregnant, including nonuse of contraception. Younger women in their early reproductive years may have a lower prevalence of contraceptive use given the desire to maintain childbearing potential compared to older women who may have already achieved their desired family size, have easier access to reproductive health information, and fewer barriers to accessing reproductive health services (37). In addition to the aforementioned reasons that may increase the likelihood of young women becoming pregnant, other pertinent issues reported from studies among young people include sexual partners prohibiting contraceptive use, negative cultural attitudes to premarital sex, and a lack of autonomy or agency by young people to consistently and correctly use contraceptive methods (22, 38, 39).

The research findings indicate that the risk of incident pregnancy was 50% lower among AGYW who consumed alcohol in the past month than those who reported not consuming alcohol. These findings were unexpected and contradicted findings from a systematic review of adolescent pregnancies in SSA that showed higher pregnancy rates among those who consumed alcohol [(40). This positive association among high-risk women under the influence of alcohol (41, 42) has been described as likely due to the lack of condom negotiation (43) and acts of intimate partner violence (44), which expose them to unintended pregnancy. Alcohol consumption by young women in our study was circumstantial as it was influenced by their engagement in transactional sex. Our findings may be explained by the fact that alcohol users in our study had a higher perceived vulnerability and therefore used contraception. Indeed, contraceptive use in our study was higher among those who consumed alcohol than those who did not.

We found that AGYW who had a higher number of sexual partners at baseline were twice as likely to get pregnant during the follow-up. This finding is corroborated by studies on young women in Tanzania (20) and school-going adolescents in South Africa (45) that showed a positive association between pregnancy and the number of sexual partners. Our findings could have the following explanations: first, volunteers who have many sexual partners have more sexual exposure, thereby increasing their likelihood of becoming pregnant; second, individuals who have many sexual partners are also likely to have other behavioral risks. Our study showed that 6 out of 10 AGYW did not use contraception and one-third were engaged in sex while drunk, which further increased the likelihood of becoming pregnant. Furthermore, a quarter of volunteers were diagnosed with either chlamydia or gonorrhea, which gave an objective measure of behavioral risk. Lastly, most of the AGYW in our study were engaged in transactional sex, with a smaller proportion identifying as sex workers. Female sex workers or women engaged in transactional sex in several settings have low condom use even when condoms are available and they have knowledge of condom use. This is attributed to low condom negotiation skills with clients (46–48), experiences of violence from clients (46, 49), and nonuse of condoms to increase their earnings from clients (47, 50), which all increase their likelihood of getting pregnant.

### 4.1. Limitations

Our study has some limitations: first, volunteers were from an urban setting and enrolled using nonrandom sampling methods that are prone to selection bias, which affects generalizability of findings. Second, we may have missed pregnancies due to the 3-month duration between study visits, likely terminated before the next visit, thereby underestimating incidence. Third, we used interviewer-administered questionnaires; data from self-reported behavior could have been affected by social desirability bias. At last, our relatively small sample size likely reduced our power to comprehensively detect associations with incident pregnancy. Despite our limitations, we recorded high retention among pregnant volunteers, as almost 72% were followed for a pregnancy outcome. Our findings contribute to the growing literature on the magnitude and factors associated with incident pregnancies among AGYW involved in transactional sex and the need to intervene in this group.

## 5. Conclusions

The incidence of unintended pregnancy in this study was high despite the provision of adolescent-friendly and free contraceptive services. Sociobehavioral studies are needed to explore individual, community-level, and health-system barriers to contraceptive utilization despite the availability of adolescent-friendly and free contraceptives. Sexual and reproductive health rights (SRHR) campaigns should create demand and motivation to use contraception among AGYW who are behaviorally vulnerable to unintended pregnancies. Given the high prevalence of STIs in this study, SRHR campaigns could also assess the acceptability of future multipurpose prevention technologies for preventing pregnancy, STIs, and HIV among young population.

## Data Availability

The anonymized raw data supporting the conclusions of this article will be made available by the authors without undue reservation, to researchers who have obtained the required approvals for data sharing.
